# A transformer-based structure-aware model for tackling the traveling salesman problem

**DOI:** 10.1371/journal.pone.0319711

**Published:** 2025-04-07

**Authors:** Chun-Sheng Zhao, Li-Pei Wong

**Affiliations:** 1 School of Computer Sciences, Universiti Sains Malaysia, Pulau Pinang, Malaysia; Shanghai Jiao Tong University - Xuhui Campus, CHINA

## Abstract

Leveraging the Transformer architecture to develop end-to-end models for addressing combinatorial optimization problems (COPs) has shown significant potential due to its exceptional performance. Nevertheless, a multitude of COPs, including the Traveling Salesman Problem (TSP), displays typical graph structure characteristics that existing Transformer-based models have not effectively utilized. Hence, this study focuses on TSP and introduces two enhancements, namely closeness centrality encoding and spatial encoding, to strengthen the Transformer encoder’s capacity to capture the structural features of TSP graphs. Furthermore, by integrating a decoding mechanism that not only emphasizes the starting and most recently visited nodes, but also leverages all previously visited nodes to capture the dynamic evolution of tour generation, a Transformer-based structure-aware model is developed for solving TSP. Employing deep reinforcement learning for training, the proposed model achieves deviation rates of 0.03%, 0.16%, and 1.13% for 20-node, 50-node, and 100-node TSPs, respectively, in comparison with the Concorde solver. It consistently surpasses classic heuristics, OR Tools, and various comparative learning-based approaches in multiple scenarios while showcasing a remarkable balance between time efficiency and solution quality. Extensive tests validate the effectiveness of the improvement mechanisms, underscore the significant impact of graph structure information on solving TSP using deep neural networks, and also reveal the scalability and limitations.

## Introduction

The combinatorial optimization problem is a specific kind of optimization problem that aims to find the optimal value within a set of discrete states. It exists widely in diverse fields, such as national security, transportation, industry, and daily life. The main approaches to solving COPs are traditional operational optimization methods, including exact methods, approximation methods, and heuristics. However, with the ongoing expansion of problem scales in practical applications and the increasing requirement for real-time answers, traditional approaches face significant computing burdens, which pose a challenge to realizing real-time solving for COPs [1–4].

Vinyals et al. [5] drew a parallel between solving COPs and the machine translation process, that is, sequence-to-sequence [6] mapping. They modified the classical encoder-decoder [7] model and proposed the Pointer Network (Ptr-Net), successfully applying deep learning to solve COPs, such as computing Delaunay triangulations, identifying planar convex hulls, and solving planar TSP. Trained with supervised learning, Ptr-Net takes the feature sequence (such as the sequence of city coordinates of the TSP) as input and directly outputs the solution (such as the visiting order of cities) through a simple forward propagation computation. Moreover, it has the ability to address any TSP that shares distributional characteristics similar to those observed in the training data. In contrast to traditional approaches, this end-to-end method does not necessitate iterative searches, exhibiting both fast solving speed and strong generalization ability. This breakthrough paved the way for a promising avenue of study within the domain of combinatorial optimization.

Since the proposal of the Ptr-Net, various innovative approaches aimed at improving the encoder, decoder, or training strategy have been introduced at top-tier conferences such as NeurIPS and ICLR in recent years. These methods have achieved outstanding optimization outcomes on COPs like TSP, the Vehicle Routing Problem (VRP), the Knapsack Problem, the Orienteering Problem (OP) and others. Bello et al. [8] adopted reinforcement learning [9] as a substitute for supervised learning to train Ptr-Net and applied it to solve the TSP and the Knapsack Problem. Their model outperformed that of Vinyals et al. As reinforcement learning tackles the difficulty of obtaining labeled samples for supervised learning in the context of COPs, it has become the dominant training approach for neural combinatorial optimization models. Since the order of data input in COPs, such as TSP and VRP, should not affect the solutions, Nazari et al. [10] simplified the Ptr-Net encoder by replacing the long short-term memory (LSTM) [11] layer with a 1D convolutional layer and significantly reduced the computational complexity of their model, saving approximately 60% training time on TSPs and outperforming classical heuristics on VRPs. Furthermore, Ma et al. [12] introduced graph neural networks (GNN) [13] to enhance the capabilities of the encoder and proposed the graph pointer network (GPN) to solve TSP and TSP with time windows. Their model exhibits notable generalizability in tackling larger-scale problem instances from synthetic datasets.

In recent years, the Transformer [14] model has achieved significant successes in the field of natural language processing (NLP) [15]. Based on the multi-head attention (MHA) [14] mechanism, the Transformer encoder exhibits a powerful capability to extract deep features from data. Given this, several latest studies have employed the Transformer to address COPs. Deudon et al. [16] used the encoder proposed in [14] to compute the feature vectors for cities in TSP. Their decoder linearly maps the cities visited by the last three steps into a query vector and utilizes the same attention mechanism as the Ptr-Net to predict the next city. Their network is trained with REINFORCE [17] in conjunction with stochastic gradient descent (SGD) with instances generated dynamically. Enhanced by a straightforward 2-opt procedure, their model achieves outcomes that are almost optimal on the TSP. Kaempfer & Wolf [18] trained their model based on the Transformer architecture to construct a fractional solution for multiple TSP and then employed beam search to convert that fractional solution into a feasible integer solution. Based on Transformer, Kool et al. [19] proposed a model that is capable of solving a wide range of COPs. They also adopted an encoder similar to the standard Transformer encoder to embed the nodes. However, they modified the decoding and training methodologies. At each decoding step, the decoder predicts the next node through an attention mechanism with a context that consists of the global representation of all cities, the first visited node and the previously visited node. Training was carried out with reinforcement learning and enhanced with the rollout baseline [19] mechanism. This model surpasses all the previously mentioned end-to-end models [5,8,10,16] on problems such as TSP, Prize Collecting TSP (PCTSP), Capacitated Vehicle Routing Problem (CVRP) and OP. Its performance approaches that of professional solvers such as Gurobi [20], LKH3 [21], and Concorde [22]. Bresson et al. [23] employed the Transformer encoder architecture to address the TSP. They modified the decoder and trained their model with reinforcement learning. Their model achieves outcomes extremely close to optimal and elevates learned heuristics to a remarkable high level.

Despite the impressive achievements of Transformer-based models, Deudon et al., Kool et al., and Bresson et al. all utilized the default Transformer encoder architecture. These models take city coordinates as sequential input and employ the Transformer’s MHA mechanism to calculate the semantic similarity between nodes. However, they do not account for the graph structure information inherent in the nodes and their relationships. Problems such as TSP and VRP exhibit typical structural characteristics of graphs. Studies conducted by Dai et al. [24], Mittal et al. [25], Li et al. [26], and others [27–30] have indicated that incorporating the structural information of graphs is beneficial for solving these problems. Therefore, we believe that properly integrating the structural information of graphs with Transformer will bolster model performance. Ying et al. [31] investigated strategies to improve the efficacy of the Transformer model within the domain of graph representation learning. They introduced three graph structure encodings (edge encoding, degree centrality encoding, and spatial encoding) to effectively encode the structural information of a graph into Transformer and proposed the Graphormer [31]. The remarkable capacity of Graphormer results in achieving state-of-the-art performance across a diverse spectrum of tasks in practical applications. Inspired by the Graphormer model, this study enhances the standard Transformer model and proposes an improved encoder architecture to capture the positional relationship features of the nodes in TSP. Furthermore, in conjunction with a modified decoding mechanism, this study proposes an end-to-end model based on the Transformer for solving TSP. Following rigorous training with reinforcement learning, the proposed model exhibits notable proficiency in tackling TSPs of various scales.

The primary contribution of this study lies in the exploration of effective strategies to leverage graph structure information to enhance the performance of Transformer-based end-to-end models in addressing TSP. Ultimately, an enhanced Transformer-based model for solving TSP is proposed, utilizing the devised encoding and decoding mechanisms. The description of the encoding and decoding mechanisms is as follows:

- Two graph encoding mechanisms are designed for the Transformer to incorporate TSP graph structure information. Specifically, the Transformer encoder is enhanced for TSP, featuring closeness centrality [32] encoding for node importance and spatial encoding for spatial relationships of TSP nodes. Empirical studies confirm the effectiveness of these designs in improving the Transformer in TSP graph modeling.
- A decoding mechanism is proposed for TSP that not only places emphasis on both the starting node and the previously visited node, but also captures the dynamic expansion of the tour by incorporating all nodes visited so far. Empirical studies demonstrate its effectiveness in improving model training and model performance.

After being trained with reinforcement learning, the proposed model achieves deviation rates of 0.03%, 0.16%, and 1.13% for TSPs of 20-node, 50-node, and 100-node, respectively, compared to the results generated by the Concorde solver, a well-established benchmark for TSP solutions.

## Preliminary

**Fig 1:**
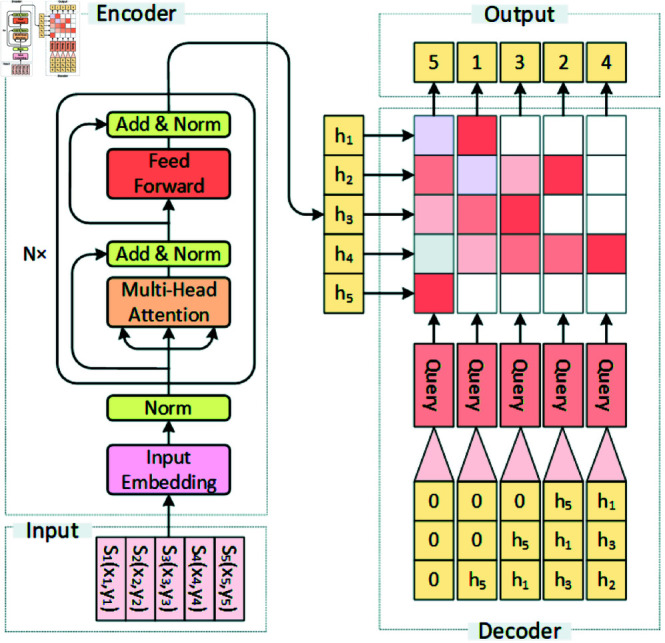
Illustration of a Transformer-based model for solving TSP.

The process of solving TSP based on the Transformer could be summarized as the mapping from an input sequence to an output sequence facilitated by a deep neural network. The model follows the classic encoder-decoder architecture and can be divided into four core modules: input, output, encoder, and decoder. Here we take the 2D Euclidean TSP as an example to introduce the basic principle. The overall architecture of the model and the solving process are illustrated in Fig 1.

**TSP:** Given a set of cities $s={\left(v_{i}(x_{i},y_{i})\right)}_{i\in [1,n]}$, the TSP involves determining the minimum cost tour $\tau (\pi_{1},\pi_{2},\ldots ,\pi_{n})$ that visits each of the *n* cities exactly once and returns to the starting city. Here, *π_k_* denotes the permutation of cities that defines their visiting order in the tour. The cost of a tour is defined as the sum of the distances traveled between consecutive cities along the route.

**Input:** Each city *v_i_* is characterized by its coordinates (*x_i_*,*y_i_*) in a 2D Euclidean space. The 2D coordinate sequence of the cities serves as the input for the model.

**Output:** Tour $\tau (\pi_{1},\pi_{2},\ldots ,\pi_{n})$.

**Encoder:** The function of the encoder is to derive a contextualized representation for each city. The attention encoder employed is similar to that used in Vaswani et al.’s Transformer [14], but the positional encoding is omitted ensuring that the node embeddings are unaffected by the input data order.

The encoder takes the 2D coordinates v_i_(*x_i_*,*y_i_*) of *n* cities as input, then computes initial *d*-dimensional (such as *d* = 128 or 256 etc.) node embeddings $h_{i}^{0}$ by means of a learned linear projection characterized by parameters *W^x^* and *b^x^*: $h_{i}^{0}=W^{x}v_{i}+b^{x}$. Following batch normalization (BN) [33], the embeddings $h_{i}^{0}$ undergo a sequence of *N* attention layers. Each attention layer comprises two sublayers: an MHA layer, responsible for facilitating message passing among nodes, and a feed-forward (FF) layer composed of two position-wise linear transformations separated by a ReLU activation. Each sublayer incorporates a skip-connection [34] and a batch normalization. The procedure is formulated as:


$$\begin{array}{ccc} \begin{array}{ccc} {ĥ}_{i}^{\ell } & =\text{BN}\left(h_{i}^{\ell -1}+\text{MHA}(h_{1}^{\ell -1},\ldots ,h_{n}^{\ell -1})\right), & \\ h_{i}^{\ell } & =\text{BN}\left({ĥ}_{i}^{\ell }+\text{FF}({ĥ}_{i}^{\ell })\right),\ell \in \{1,\ldots ,N\}. \end{array} & & \end{array}$$ (1)

*ℓ* indicates the attention layer index. The output of the previous attention layer serves as the input of the next encoding layer. Current studies such as [16,19] prefer to employ *N* = 3 attention layers and hidden layer dimensions of 512, aiming to strike a favorable balance between high-quality results and computational efficiency.

The MHA sublayer performs self-attention computations across *M* distinct learned subspaces, thus earning its designation as “multi-head”. This process yields *M* representations (each with dimensionality *d* ∕ *M*) for each city. Within each head, the representation of each city is derived as a weighted aggregation of city embeddings, with the weights determined by an affinity function operating on queries *Q* and keys *K* associated with the cities. Subsequently, these representations are concatenated and projected back to a *d*-dimensional representation. The self-attention mechanism is formally defined as


$$\begin{array}{ccc} Attention(Q;K;V)=softmax(\frac{QK^{T}}{\sqrt{d\;}})V, & & \end{array}$$ (2)

where *Q*=*W^Q^H*, *K*=*W^K^H*, *V*=*W^V^H*, and $H=[h_{1},h_{2},\ldots ,h_{n}]$. The matrices *W^Q^*, *W^K^*, and *W^V^* are all parameters that should be learned through training.

**Decoder:** The role of the decoder is to utilize an attention mechanism to progressively construct a solution in an autoregressive manner. Autoregressive means that at each step the previously generated symbol is used as an additional input to generate the next until a complete solution is constructed.

Given the presence of several effective decoding mechanisms [16,19,23], here we take the mechanism proposed by Deudon et al. [16] as an illustration, which is shown in Fig 1. At each decoding step *t*, the last three visited cities are aggregated into a query vector *q_t_*:


$$\begin{array}{ccc} q_{t}=ReLu\left(W_{1}h_{\pi (t-1)}+W_{2}h_{\pi (t-2)}+W_{3}h_{\pi (t-3)}\right)\in {\mathbb{R}}^{d^{\prime }}. & & \end{array}$$ (3)

*W*_1_, *W*_2_, and *W*_3_ are learnable parameters. The query *q_t_* interacts with the encoder output to calculate a probability distribution $p_{\theta }(\pi_{t}|s,\pi_{1:t-1})$ to identify the city to visit next. This pointing mechanism is parameterized by three crucial factors: two attention matrices labeled *W_ref_* and *W_q_*, alongside an attention vector denoted by *v*. The formulation is articulated as follows:


uit= {vT tanh ⁡ (Wrefhi+Wqqt)if i∉{π1,…,πt−1}−∞otherwise, (4)

$$\begin{array}{ccc} p_{\theta }(\pi_{t}|s,\pi_{1:t-1})=softmax\left(C\;\tanh(u^{t}∕T)\right). & & \end{array}$$ (5)

A mask is employed to assign a value of  − *∞* to the log-probabilities associated with cities already visited within the tour, as elucidated by Eq (4). This operation is essential to ensure that the decoder generates admissible permutations of the input. Subsequently, the logits undergo a bounding operation within the interval  [ − *C* , + *C* ]  to regulate entropy, while a temperature hyperparameter *T* is introduced to govern the level of confidence in the sampling process, as shown in Eq (5). According to the pointing probabilities $p_{\theta }(\pi_{t}|s,\pi_{1:t-1})$, the next city is selected by sampling. Upon selection of the subsequent city, the query *q*_*t*+1_ will be revised to include the selected city, and the autoregressive process will be completed upon completion of the tour.

## Proposed model

In this study, adhering to the classical encoder-decoder structure, a model tailored for 2D Euclidean TSP is proposed based on the Transformer. For a TSP instance denoted as *s*, which represents a fully connected graph comprising *n* nodes, the encoder maps the input set $s=(v_{1},v_{2},\ldots ,v_{n})$ into a set of continuous representations $h=(h_{1},h_{2},\ldots ,h_{n})$. Given *h*, the decoder then autoregressively generates a permutation $\tau =(\pi_{1},\pi_{2},\ldots ,\pi_{n})$ of the input *s*, which serves as the final solution.

### Encoder

**Fig 2:**
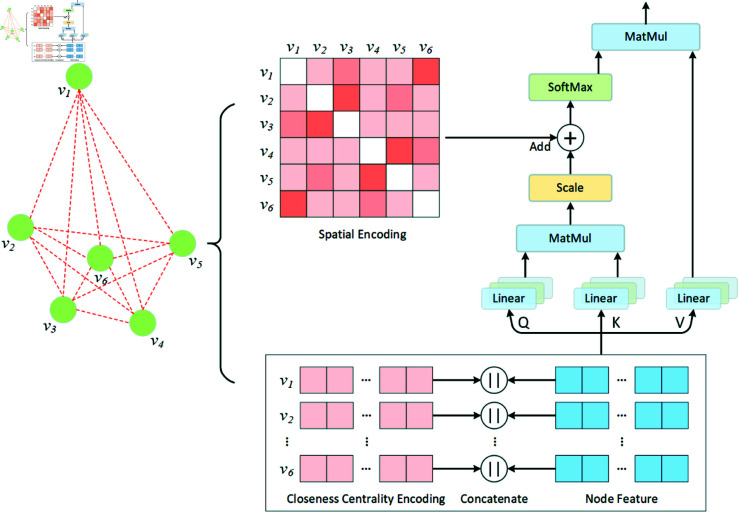
Illustration of the proposed closeness centrality encoding and spatial encoding for enhancing the Transformer encoder.

Our encoder inherits the overall structure of the encoder depicted in Fig 1. However, as emphasized in our introduction, there exists a potential in developing methodologies to exploit the structural information inherent in TSP graphs. Therefore, two effective encoding modules are introduced into our encoder to enhance its structure-aware ability. The mechanisms are illustrated in Fig 2.

#### Closeness centrality encoding.

In contemporary Transformer-based models, the computation of the attention distribution is predominantly based on semantic correlations among nodes, neglecting the insightful information offered by node centrality. Node centrality is a fundamental metric that indicates the importance of a node within a graph. The Graphormer incorporates the classical degree centrality [31] as an additional signal to enhance the Transformer. As this model focuses on fully connected TSPs, where all nodes have the same degree, degree centrality is no longer a significant feature. Consequently, closeness centrality is adopted as an alternative metric in this study to measure the importance of each node and is integrated into the Transformer. More specifically, a centrality encoding mechanism is developed that assigns a real-valued embedding vector *z* to each node based on its closeness centrality. This vector is then concatenated with the embedding of the node’s coordinates, constructing the initial input vector $h_{i}^{0}$ of the encoder:


$$\begin{array}{ccc} \begin{array}{cc} h_{i}^{0} & =[x_{i},y_{i}]\;||\;z_{cen(v_{i})}, \end{array} & & \end{array}$$ (6)

where $[x_{i},y_{i}]\in {\mathbb{R}}^{d}$ represents the embedding of the coordinates (*x_i_,y_i_*), $z_{cen(v_{i})}\in {\mathbb{R}}^{d}$ denotes the learnable embedding vector associated with the closeness centrality *cen*(*v_i_*), and  | |  represents the concatenation operation. Integrating centrality encoding into the input allows the attention mechanism to effectively capture signals indicating node importance in both keys and queries. This integration empowers the model to simultaneously capture semantic correlations and prioritize node importance within the attention mechanism.

#### Spatial encoding.

The conventional Transformer [14] model incorporates positional encodings into input embeddings to leverage relative or absolute position information from sequential data tokens. However, this approach is not directly applicable to TSP. Unlike typical sequential data, where tokens are arranged in a linear fashion, TSP instances involve nodes situated in a 2D spatial space. These nodes are interconnected by edges, forming a graph structure that deviates from the linear sequence assumed by the conventional Transformer model. The input order of the TSP nodes does not accurately reflect their true positional relationships. As a result, when applying the Transformer to solve TSP, researchers commonly eliminate the positional encoding module to mitigate the influence of the input order of the nodes on final solutions. However, this approach neglects the spatial positional relationships between nodes, preventing models from leveraging this valuable information. In the TSP context, the spatial arrangement of the nodes directly affects the travel distances between the nodes, which is a critical factor in scouting the shortest feasible path. Nodes in close spatial proximity are more likely to be connected by edges within the solution. Recognizing this, spatial information becomes vital for various heuristic algorithms [35] and optimization techniques [36,37], aiding in the search for the shortest tour. To incorporate the structure information of the TSP nodes into our model, the Graphormer spatial encoding mechanism is employed. When applied to a given graph *G*, Graphormer defines a function $\phi (v_{i},v_{j}):V\times V\to \mathbb{R}$ that quantifies the spatial relationship between the nodes *v_i_* and *v_j_* within the graph *G*. This function *ϕ* is typically defined based on the connectivity between the nodes in the graph. For this study, *ϕ*(*v_i_,v_j_*) is specifically defined as the Euclidean distance between nodes *v_i_* and *v_j_*. Each output value is then assigned a learnable scalar that functions as a bias term within the self-attention module. The  ( *i* , *j* ) -element of the attention query-key product matrix *A* at Transformer encoding layer *ℓ* is denoted as $A_{ij}^{\left(\ell \right)}$ and is formulated as


$$\begin{array}{ccc} A_{ij}^{\left(\ell \right)}=\frac{\left(W_{\left(\ell \right)}^{Q}h_{i}^{\left(\ell -1\right)}\right){\left(W_{\left(\ell \right)}^{K}h_{j}^{\left(\ell -1\right)}\right)}^{T}}{\sqrt{d\;}}+b_{\phi (v_{i},v_{j})}. & & \end{array}$$ (7)

The $b_{\phi (v_{i},v_{j})}$ represents a learnable scalar indexed by $\phi (v_{i},v_{j})$. It is shared across all our Transformer encoding layers. The utilization of $b_{\phi (v_{i},v_{j})}$ enables each node within each encoding layer to dynamically adjust its attention to other nodes based on the structural information of the graph. For example, if $b_{\phi (v_{i},v_{j})}$ is learned to be inversely related to the value of $\phi (v_{i},v_{j})$, the model will tend to allocate greater attention to the nearby nodes while diminishing attention to those farther away.

Following the encoding process delineated in Fig 1, our encoder receives the expanded embeddings $h_{i}^{0}$, which encapsulate centrality information of nodes, and executes multiple layers of MHA encoding employing the attention mechanism enhanced by spatial encoding. This process ultimately yields node encodings with graph structure features.

### Decoder

The decoding process unfolds sequentially. At each time step *t* ∈ { 1 , 2 , *…* , *n* } , the decoder produces an output node *π_t_* leveraging both the encoding results generated by the encoder and the partial tour *π*1:*t*−1. The decoding mechanism adopted in this study is similar to the approach proposed by Kool et al. [19]. Specifically, it introduces a designated context node, denoted *c*, to encapsulate the decoding context. This context node is subsequently employed as a query, while the encoder output serves as reference, thereby facilitating the prediction of the probability distribution of the next node through a single-head attention (SHA). To enhance model performance, an additional attention layer for the context node, named glimpse [5,8], is incorporated atop the encoder. This layer aggregates contributions from various nodes in the input sequence, contributing to overall model improvement.

**Fig 3:**
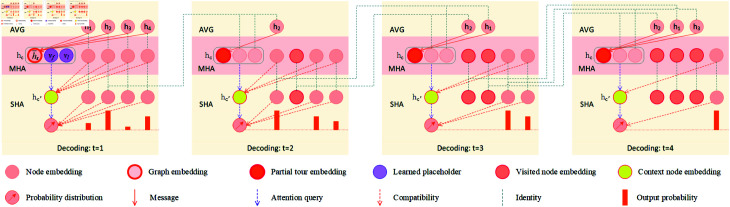
Decoding procedure of the TSP problem. This example illustrates how a tour *π* = ( 2 , 1 , 3 , 4 )  is generated based on the embedding results (*h*_1_,*h*_2_,*h*_3_,*h*_4_) obtained from the encoder. Best viewed in color.

Our strategy for constructing the context node *c* differs from that of Kool et al. They utilize the mean of the encoding of all nodes as a graph token and combine it with the starting node and the previously selected node to construct the context node. For TSP, since the newly selected node *π_t_* must be directly linked to the previously chosen node *π*_*t*−1_ to form a tour, each decoding step is highly dependent on the previously visited node *π*_*t*−1_. In addition, the path must return to the starting point (the node visited in the first step). Therefore, including the starting node and the previously selected node in the context node to ensure continuous attention to these critical nodes is an effective mechanism [38,39]. This study adopts this mechanism in the construction of the context node. However, the graph token utilized by Kool et al. encapsulates global features of the input sequence and remains static throughout the entire decoding process. This static nature of the graph token limits its ability to capture dynamic changes in the decoding context [39]. Specifically, it overlooks the influence of other nodes, such as visited nodes and candidate nodes, on the decoding decisions. Luo et al. [38] addressed a similar issue in their research by utilizing the starting node, the destination node, and the available nodes, thus achieving dynamic learning and enabling their model to adjust and refine the relationships between the starting/destination nodes and the available nodes. The available nodes employed in [38] consist of a set of unvisited nodes. In fact, the impact of visited nodes on the decoding process is also significant and has been emphasized in multiple studies, such as those of Bello et al. [8], Deudon et al. [16], and Bresson et al. [23]. Therefore, this study calculates the mean of the encodings of all visited nodes to create a token representing the latest partial tour, which is different from the mechanism employed in [38]. This token is then combined with the nodes visited in the first and previous steps to construct the context node for our decoder. At each time step, our context node not only emphasizes the significance of both the first and the most recently visited nodes, but also conveys information regarding all previously visited nodes to the decoder. A more detailed illustration of the decoding process is illustrated in Fig 3.

#### Constructing the context node.

For *t* = 1, we calculate the mean of the encodings of all nodes and obtain the graph token ${\bar{h}}_{g}$. The graph token combines with two learnable *d*-dimensional placeholders *V_f_* and *V_l_*, yielding context node $h_{c}^{1}$. This context node is employed to launch the decoder to predict the starting node. For *t* > 1, context node $h_{c}^{t}$ is built with the embeddings of the first visited node *h*_*π*1_, the previously visited node *h*_*π*_*t*−1__, and the mean of all visited nodes ${\bar{h}}_{t}$:


$$\begin{array}{ccc} \begin{array}{ccc} {\bar{h}}_{g} & =\text{mean}(h_{1},h_{2},\ldots ,h_{n}), & \\ {\bar{h}}_{t} & =\text{mean}(h_{\pi_{1}},h_{\pi_{2}},\ldots ,h_{\pi_{t-1}}),\\ h_{c}^{t} & =\{\begin{array}{cc} {}[{\bar{h}}_{g},V_{f},V_{l}]\; & t=1\\ {}[{\bar{h}}_{t},h_{\pi_{1}},h_{\pi_{t-1}}]\; & t>1. \end{array} \end{array} & & \end{array}$$ (8)

Here,  [ ⋅ , ⋅ , ⋅ ]  denotes the horizontal concatenation operator.

#### Glimpsing with MHA.

At each decoding step *t*, a single glimpse using *M*-head attention is performed to compute a context node encoding $h_{c^{\prime }}^{t}$. This mechanism is inspired by the work of Bello et al. [8]. For each head, the attention keys and values are derived from the node encodings *h_i_* generated by our encoder, and the query $q_{c}^{t}$ is generated from the context node $h_{c}^{t}$:


$$\begin{array}{ccc} \begin{array}{ccc} q_{c}^{t}=W_{g}^{Q}h_{c}^{t},k_{i}=W_{g}^{K}h_{i},v_{i}=W_{g}^{V}h_{i} & & i\in [1,n]. \end{array} & & \end{array}$$ (9)

$W_{g}^{Q},W_{g}^{K}$, and $W_{g}^{V}$ are all learnable parameters. At time *t*, the compatibility $u_{c}^{t}$ between the query $q_{c}^{t}$ and all nodes is calculated as follows:


$$\begin{array}{ccc} u_{ci}^{t}=\{\begin{array}{c} \begin{array}{ccccc} \frac{k_{i}^{T}q_{c}^{t}}{\sqrt{d_{k}}} & & \text{if}\;i\neq \pi_{t^{\prime }}, & \forall t^{\prime }<t & \\ -\infty & & \text{otherwise}. \end{array}\; \end{array} & & \end{array}$$ (10)

Here, $u_{ci}^{t}$ denotes the compatibility score between $q_{c}^{t}$ and node *i* at time *t*, and *d_k_*=*d∕M* is the dimensionality of the keys and queries. The assignment of $u_{ci}^{t}=-\infty$ is utilized to mask nodes that have already been visited. Based on compatibilities $u_{ci}^{t}$, the attention weights $a_{ci}^{t}\in [0,1]$ are derived through a softmax. For each head *m* ∈ [ 1 , *M* ] , the vector $h_{c_{m}}^{t}$ received by context node $h_{c^{\prime }}^{t}$ is generated as a weighted combination of values *v_i_*, denoted by:


$$\begin{array}{ccc} h_{c_{m}}^{t}=\overset{n}{\underset{i=1}{\sum }}a_{ci}^{t}v_{i}. & & \end{array}$$ (11)

Finally, a single *d*-dimensional context vector $h_{c^{\prime }}^{t}$ is obtained by projecting these vectors utilizing parameter matrices $W_{m}^{O}$:


$$\begin{array}{ccc} h_{c^{\prime }}^{t}=\overset{M}{\underset{m=1}{\sum }}W_{m}^{O}h_{c_{m}}^{t}. & & \end{array}$$ (12)

#### Calculating probabilities.

The probabilities $p_{\theta }(\pi_{t}|s,\pi_{1:t-1})$ are calculated by an independent decoder layer equipped with a single-head attention. Firstly, the compatibility $u_{c^{\prime }i}^{t}$ is computed according to the context vector $h_{c^{\prime }}^{t}$. Then, following Bello et al. [8], a tanh clipping is applied to the results (prior to masking) to constrain them within the range  [ − *c* , *c* ] :


$$\begin{array}{ccc} u_{c^{\prime }i}^{t}=\{\begin{array}{c} \begin{array}{ccccc} & C\cdot \tanh\left(\frac{k_{i}^{T}h_{c^{\prime }}^{t}}{\sqrt{d_{k}}}\right) & & \text{if }i\neq \pi_{t^{\prime }},\forall t^{\prime }<t & \\ & -\infty & & \text{otherwise}. \end{array}\; \end{array} & & \end{array}$$ (13)

These compatibilities are interpreted as unnormalized log-probabilities. The ultimate selection probability of node *i* at time step *t*, denoted as $p_{i}^{t}$, is computed using the softmax function:


$$\begin{array}{ccc} p_{i}^{t}=p_{\theta }(\pi_{t}=i|s,\pi_{1:t-1})=\frac{e^{u_{c^{\prime }i}^{t}}}{\begin{array}{c} \overset{n}{\underset{j=1}{\sum }}e^{u_{c^{\prime }j}^{t}} \end{array}}. & & \end{array}$$ (14)

Finally, based on the probability distribution, the decoder employs a greedy or sampling strategy to select a subsequent node to visit.

## Training the model

Following studies [8,16,19,23], a model-free policy-based reinforcement learning approach is employed to refine the parameters (denoted as *θ*) of our neural network. The deep neural network developed in this study defines a stochastic policy $p_{\theta }(\tau \;\;|\;\;s)$ that maps the input city sequence *s* to the output sequence *τ*. At step *t*, considering the visited cities *π*_1:*t*−1_ and the city sequence *s*, the probability of the subsequent city *π_t_* is $p_{\theta }(\pi_{t}\;|\;s,\pi_{1:t-1})$. Utilizing the chain rule, the probability of an entire tour *τ* can be deduced as:


$$\begin{array}{ccc} p_{\theta }(\tau \;|\;s)=\overset{n}{\underset{t=1}{\prod }}p_{\theta }(\pi_{t}\;|\;s,\pi_{1:t-1}). & & \end{array}$$ (15)

Given an input set of cities *s*, the fundamental concept involves assigning elevated probabilities to preferred tours while minimizing probabilities for less desirable ones. As the quality of a tour is evaluated by its length *L* ( *τ* )  (which is endeavored to minimize), for a given problem instance *s*, the training objective is defined as the expected tour length:


$$\begin{array}{ccc} J(\theta \;|\;s)=E_{\tau ∼p_{\theta }(\cdot |s)}L(\tau \;|\;s). & & \end{array}$$ (16)

To endow the policy $p_{\theta }(\tau \;|\;s)$ with generalization capabilities, the training instances should be sampled from a distribution *S*. Consequently, the overarching training objective is formulated as:


$$\begin{array}{ccc} J(\theta )=E_{s∼S}J(\theta \;|\;s). & & \end{array}$$ (17)

The policy gradient method along with SGD is adopted to iteratively update the model parameters. The gradient of Eq (17) could be formulated using the established REINFORCE algorithm [17] as:


$$\begin{array}{ccc} \nabla_{\theta }J(\theta \;|\;s)=E_{\tau ∼p_{\theta }(\cdot |s)}\left[\left(L(\tau \;|\;s)-b(s))\nabla_{\theta }logp_{\theta }(\tau \;|\;s\right)\right], & & \end{array}$$ (18)

where *b*(*s*) represents a baseline function utilized to estimate the anticipated tour length of the input sequence *s*, and *L* ( *τ* ∣ *s* ) − *b* ( *s* )  determines the direction of gradient updates.

By drawing *B* *i.i.d.* sample instances $s_{1},s_{2},\ldots ,s_{B}∼S$ and sampling one solution for each, *i.e.* $\tau_{i}∼p_{\theta }(\cdot \;|\;s_{i})$, the gradient of Eq (18) could be approximated by Monte Carlo sampling as follows:


$$\begin{array}{ccc} \nabla_{\theta }J(\theta )\approx \frac{1}{B}\overset{B}{\underset{i=1}{\sum }}\left(L(\tau_{i}\;|\;s_{i})-b(s_{i})\right)\nabla_{\theta }logp_{\theta }(\tau_{i}\;|\;s_{i}). & & \end{array}$$ (19)

A robust baseline *b*(*s*) is essential to mitigate the variance in the gradient and accelerate the training process. In this study, the greedy rollout baseline method introduced by Kool et al. [19] is adopted as a substitute for employing an additional critic network to approximate *b*(*s*) to improve training. For each training epoch, following a methodology akin to the approach employed in the deep Q-network (DQN) [40] where the target Q-network is frozen after each training epoch, the best policy achieved up to the current epoch is preserved as the baseline policy denoted as $p_{\theta_{BL}}$. The baseline *b*(*s*) estimates the tour length $L(\tau^{BL})$ of the solution *τ^BL^*, which is derived from the deterministic greedy rollout of the baseline policy. If the current training policy *p_θ_* surpasses the baseline, the tour length *L* ( *τ* )  of the sampled solution *τ* based on *p_θ_* will be shorter than *b*(*s*), and *L* ( *τ* ) − *b* ( *s* )  is negative, resulting in the current policy being reinforced, and vice versa. After the training of each epoch, the performance of both the new policy and the baseline is compared on a validation dataset with a size of 10,000 instances. If the new policy outperforms the baseline and the enhancement demonstrates statistical significance determined by a paired t-test [41] with a predetermined significance level of *α* = 5*%*, the baseline parameters *θ_BL_* are updated with newly learned parameters *θ*. As a result, this approach ensures that our model is consistently challenged by the most superior model achieved thus far, aligning with the self-critical training principles proposed by Rennie et al. [42]. To prevent overfitting, once the baseline is updated, the validation dataset is refreshed. As the baseline model periodically updates and saves the best model, the latest baseline model is employed as the final output model. The algorithmic procedure can be located in Algorithm 1 as detailed in reference [19].

## Experiments

This study focuses on the TSP and systematically evaluates the efficacy of the proposed model across three benchmark tasks: Euclidean TSP20, TSP50, and TSP100. The experiments are implemented using Ubuntu 20.04, Python 3.8, and PyTorch 1.11.0. Consistent hyperparameters are employed for all problems.

### Hyperparameters

For all three problems, all datasets are uniformly sampled from the 2D unit square [0,1]^2^. The size of the training dataset is 1,280,000, while the validation and test datasets each consist of 10,000 instances. The mini-batch size is defined as 512; thus, the parameters are updated 2,500 times per epoch. Early stopping criteria are applied with a threshold set at 20 epochs. The two coordinates and the closeness centrality of each city are individually embedded in 128-dimensional spaces and then concatenated as a *d* = 256 combined embedding. The attentive encoder consists of 3 stacks, each equipped with *M* = 8 parallel heads, and the hidden dimensionality is *d* = 256. For each head, the dimensionality is specified as $d_{h}=d∕M=32$. Both the input and output dimensions of the feed-forward sublayer are configured to be 256, featuring an inner layer with a dimensionality of 512 and employing a ReLu activation. The pointing mechanism operates within a 256-dimensional space. Model parameters *θ* are initialized according to a uniform random distribution within the interval of $\left(-1∕\sqrt{d\;},1∕\sqrt{d\;}\right)$. The tanh logits for the pointing mechanism are clipped to the interval  [ − 10 , 10 ] . Adam [43] is utilized as the optimization algorithm in conjunction with SGD. The learning rate $\eta =10^{-4}$ is fixed for the first 100 epochs to speed up initial learning and then decayed every 2 epochs by 0.98. In the first epoch, an exponential baseline  ( *β* = 0 . 8 )  is adopted as the rollout baseline to warmup the learning. The significance threshold is set to *α* = 5*%* for the paired t-test during the selection of the baseline.

### Training

**Fig 4:**
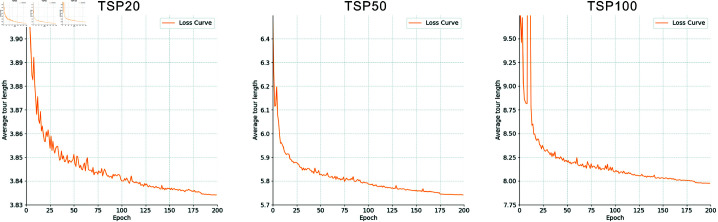
Learning progression of the proposed model over time on TSP20, TSP50, and TSP100.

The proposed model is trained individually to solve TSP20, TSP50, and TSP100. Each training session is conducted exclusively on a single NVIDIA GeForce RTX 3090 (24G) graphics card, with 200 training epochs. The average duration of each epoch is 217, 525, and 1,330 seconds for TSP20, TSP50, and TSP100, respectively. Following each epoch of training, the learned policy is evaluated using a validation dataset, employing the greedy mechanism. The loss is quantified as the mean tour length of the validation dataset. The evolution of our model’s learning progress over time on problems of different sizes is illustrated in Fig 4.

As shown in the figure, the model exhibits relatively consistent convergence trends and processes across problems of different scales. Initially, each model starts with a higher loss. As training progresses, the losses decrease steadily, converging towards stable minima. This trend indicates that the models are effectively exploring and refining their policies. The curves show a sharp decline before they begin to plateau, suggesting that our models rapidly comprehend and adapt to the complexities of different TSP instances. Additionally, while minor irregularities are observed, the consistent progression towards lower losses confirms the effectiveness of the training methodology.

## Results and discussion

The performance of the proposed model in tackling TSP20, TSP50, and TSP100 is assessed through the mean tour length of datasets consisting of 10,000 distinct test instances for each problem size, respectively. The test datasets are constructed in accordance with the methodology adopted by Kool et al. [19] and serve as the benchmark for our comparative study. During inference, two distinct decoding strategies are utilized. The first strategy, known as greedy decoding, sequentially selects the city with the highest probability during decoding, generating a single solution. The second strategy, called sampling, generates 1,024 candidate solutions for each problem instance, from which the best solution is selected. The performance of the proposed model under greedy decoding is systematically contrasted with baselines that generate singular solutions. Simultaneously, the performance of the proposed model employing the sampling strategy is rigorously evaluated against baselines that employ sampling or search methodologies to explore multiple solutions. For each problem instance, the result obtained by Concorde is reported as the benchmark for calculating the optimality gaps, which are expressed as a percentage. Furthermore, the approaches compared with ours are categorized into two groups: non-Transformer-based and Transformer-based.

### Comparison with non-Transformer-based methods

Non-Transformer-based comparative methods include: (1) classic heuristics, including nearest neighbor, nearest insertion, random insertion, farthest insertion, and Clarke Wright savings [44] algorithms; these algorithms are non-learned baseline algorithms that construct a solution sequentially, resembling the structure of the proposed model; (2) OR Tools, a powerful solver developed by Google for routing problems; (3) deep learning methods grounded in construction and improvement heuristics, involving both reinforcement [8,24] and supervised [5,28] learning methods. We implemented the classic heuristics in Python. For OR Tools, we develop a Python script specifically for solving TSP, adhering to the TSP-solving guidelines provided by OR Tools and adopting its constraint solver. We utilize default search parameters, and the initial solution strategy is configured to PATH_CHEAPEST_ARC. All implementations are evaluated on our test datasets. Their optimal gaps are computed on the basis of the results of Concorde. For deep learning methods proposed by Bello et al. [8] and Joshi et al. [28], we refer directly to the comprehensive experimental results provided in their original studies. Meanwhile, the experimental results for the models introduced by Vinyals et al. [5] and Dai et al. [24] are obtained from the study by Kool et al.’s [19].

Table 1 clearly illustrates the efficacy of the proposed model employing the greedy decoding strategy to solve TSPs of various sizes. The model achieves an optimality gap of only 0.10% on TSP20, closely approximating the performance of the Concorde solver. This gap increases modestly to 0.81% for TSP50 and to 2.72% for TSP100, demonstrating strong performance even as the complexity of the problem increases. Compared with classic heuristic methods, such as nearest neighbor, nearest insertion, random insertion, and farthest insertion, the proposed model demonstrates significant advancements in solution quality. Among these methods, the farthest insertion achieves the best results on TSP20 and TSP50, with optimality gaps of 2.53% and 7.29%, respectively, while random insertion performs the best on TSP100, achieving a gap of 9.62%. The proposed model surpasses these results with reductions of 2.43%, 6.48%, and 6.90% on TSP20, TSP50, and TSP100, respectively, underscoring its ability to outperform the most commonly applied traditional heuristics. The comparison with the Clarke Wright savings algorithm, a more advanced and effective heuristic, further highlights the model’s effectiveness. Clarke Wright savings achieve optimality gaps of 3.78%, 6.65%, and 8.27% for TSP20, TSP50, and TSP100, respectively. The proposed model improves upon these results by 3.68%, 5.84% and 5.55%, demonstrating its capability to address both general and advanced heuristic approaches. The OR Tools solver also delivers competitive results with optimality gaps of 0.73%, 2.67%, and 3.75% for TSP20, TSP50, and TSP100. Despite its strong performance, the proposed model achieves lower gaps, reducing optimality gaps by 0.63% on TSP20, 1.86% on TSP50 and 1.03% on TSP100. Finally, compared to non-Transformer-based deep learning methods that greedily construct solutions, the proposed model consistently outperforms state-of-the-art alternatives, including those proposed by Vinyals et al., Bello et al., Dai et al., and Joshi et al. Among these comparative methods, Joshi et al. achieve the smallest optimality gaps of 0.60% and 3.10% on TSP20 and TSP50, while Bello et al. perform best on TSP100 with an optimality gap of 6.90%. However, the proposed model outperforms these approaches in all three problem sizes, reducing the optimality gaps by 0.50%, 2.29%, and 4.18%, respectively.

From the perspective of computational time, the proposed model demonstrates competitive efficiency when executed in greedy mode. Specifically, it requires 0.016, 0.037, and 0.076 seconds to solve TSP20, TSP50, and TSP100 instances, respectively. Compared to classic heuristic methods, such as nearest neighbor, nearest insertion, random insertion, and farthest insertion, the proposed model generally consumes slightly more time. However, this marginal increase is offset by substantial improvements in solution quality. In particular, as the problem size increases, the efficiency gap between the proposed model and these traditional heuristic methods narrows. For example, the model solves a single TSP100 problem in 0.076 seconds, which is faster than the 0.082 seconds required by random insertion and the 0.103 seconds by farthest insertion. When compared with Clarke Wright savings and OR Tools, the proposed model demonstrates significantly superior efficiency on TSP50 and TSP100, while consistently achieving improved solution quality across all problem scales. Unfortunately, for non-Transformer-based deep learning methods, a direct and fair comparison is not possible because some studies (Vinyals et al. and Dai et al.) do not report their time data, while others (Bello et al. and Joshi et al.) do record time data, but these are based on differing software and hardware platforms. Nevertheless, despite these limitations, the available results reveal notable trends. Bello et al.’s model demonstrates the best computational efficiency. However, its solution quality remains significantly inferior to that of the proposed model. Compared to Joshi et al., even when accounting for platform differences, the computational efficiency of the proposed model is on a completely different level, solving instances several orders of magnitude faster.

**Table 1:** Performance comparison of the proposed model with classic heuristics, OR Tools, and non-Transformer-based deep learning models using greedy strategy on TSP20, TSP50, and TSP100. Obj represents the objective, which refers to the tour length. The optimality gaps (%) are computed with respect to Concorde. In the “Type” column, the abbreviations denote the following methodologies: H for heuristic, SL for Supervised Learning, RL for Reinforcement Learning, Ptr-Net for Pointer Network, and GCN for Graph Convolutional Network. The “–” symbol indicates that the corresponding data are not available due to their absence in the original studies.

Method	Type	TSP20			TSP50			TSP100		
		Obj.	Gap (%)	Time (s)	Obj.	Gap (%)	Time (s)	Obj.	Gap (%)	Time (s)
*Concorde*	Solver	3.836	0.00	0.022	5.696	0.00	0.057	7.765	0.00	0.290
*Nearest neighbor*	H	4.497	17.23	**0.000**	7.003	22.95	**0.002**	9.684	24.71	0.014
*Nearest insertion*	H	4.331	12.90	0.002	6.780	19.03	0.016	9.459	21.82	0.082
*Random insertion*	H	4.007	4.46	**0.000**	6.131	7.64	**0.002**	8.512	9.62	**0.008**
*Farthest insertion*	H	3.933	2.53	0.001	6.111	7.29	0.015	8.703	12.08	0.103
*Clarke Wright savings*	H	3.981	3.78	0.003	6.075	6.65	0.044	8.407	8.27	0.508
*OR Tools*	H	3.864	0.73	0.022	5.848	2.67	0.173	8.056	3.75	0.872
*Vinyals et al.* [19]	Ptr-Net+SL	3.880*	1.15*	–	7.660*	34.48*	–	–	–	–
*Bello et al.* [8]	Ptr-Net+RL	3.890*	1.42*	–	5.950*	4.46*	0.003*	8.300*	6.90*	0.010*
*Dai et al.* [19]	Structure2vec+RL	3.890*	1.42*	–	5.990*	5.16*	–	8.310*	7.03*	–
*Joshi et al.* [28]	GCN+SL	3.860*	0.60*	6.000*	5.870*	3.10*	55.000*	8.410*	8.38*	360.000*
*Proposed model*	Transformer+RL	**3.840**	**0.10**	0.016	**5.742**	**0.81**	0.037	**7.976**	**2.72**	0.076

* Data are sourced from the cited references.

Table 2 presents a comparative analysis of different approaches that utilize a sampling strategy to solve TSPs across three different problem sizes. Sampling is a widely employed technique for improving solution quality. By conducting 1,024 samples, the proposed model further reduces the optimality gap to 0.03% on TSP20, 0.16% on TSP50, and 1.13% on TSP100. These results demonstrate the outstanding capability of the proposed model in achieving near-optimal solutions on various problem scales. Although sampling improves the performance of OR Tools over its greedy mode, the proposed model still markedly outperforms it, achieving optimality gap reduction of 0.34%, 1.67%, and 1.77% for TSP20, TSP50, and TSP100, respectively. When compared to Joshi et al.’s model augmented with beam search, our model demonstrates consistently and significantly enhanced solution quality across TSP20, TSP50, and TSP100. For instance, on TSP100, where Joshi et al.’s model achieves an optimality gap of 2.11%, the proposed model achieves a reduced gap of 1.13%, indicating an improvement of 0.98%. Similarly, when evaluated against the approaches of Bello et al.’s and Costa et al.’s, the proposed model achieves consistently lower optimality gaps on TSP50 and TSP100, while exhibiting only marginal differences on TSP20. It is important to note that while Bello et al. report a lower objective value of 5.7 on TSP50, this does not translate to superior performance due to differences in the datasets used. Such variability prevents a direct comparison of objective values. However, based on the optimality gaps, which provide a reliable measure of performance, the proposed model outperforms Bello et al. on TSP50.

**Table 2:** Performance comparison of the proposed model with OR Tools and non-Transformer-based deep learning models using sampling strategy on TSP20, TSP50 and TSP100. Obj represents the objective, which refers to the tour length. The optimality gaps (%) are computed with respect to Concorde. In the “Type” column, the abbreviations denote the following methodologies: S for Sampling, S (#) for sampling conducted # times, BS for Beam Search, BS (#) for Beam Search with a beam width of #, and RNN for Recurrent Neural Network. The “–” symbol indicates that the corresponding data are not available due to their absence in the original studies.

Method	Type	TSP20			TSP50			TSP100		
		Obj.	Gap (%)	Time (s)	Obj.	Gap (%)	Time (s)	Obj.	Gap (%)	Time (s)
*Concorde*	Solver	3.836	0.00	0.022	5.696	0.00	0.057	7.765	0.00	0.290
*OR Tools* [19]	H, S (1,024)	3.850*	0.37*	20.604	5.800*	1.83*	163.074	7.990*	2.90*	803.508
*Bello et al.* [8]	Ptr-Net+RL, S (1,280,000)	**3.820** *	**0.00** *	–	**5.700** *	0.35*	1,080.000*	7.880*	1.42*	2,640.000*
*Joshi et al.* [28]	GCN+SL, BS (1,280)	3.840*	0.10*	20.000*	5.710*	0.26*	120.000*	7.920*	2.11*	600.000*
*Costa et al.* [45]	GCN+RNN+RL, S (1,000)	3.840*	**0.00** *	600.000*	5.710*	0.21*	780.000*	7.860*	1.26*	1,260.000*
*Proposed model*	Transformer+RL, S (1,024)	3.837	0.03	17.732	5.705	**0.16**	41.050	**7.853**	**1.13**	82.561

* Data are sourced from the cited references. For OR Tools, the objective values and optimality gaps are taken from Kool et al. [19], as reproducing these results with 1,024 samples is computationally prohibitive due to the large number of instances in the test datasets. To estimate the computational time, we solve 10 instances for each scale of TSP, with each instance sampling 1,024 solutions. Each sample starts from a randomly initialized node. The reported time is the average computational time across the 10 instances for each TSP scale.

In addition to achieving outstanding solution quality, the proposed model demonstrates competitive efficiency when executed in sampling mode. Specifically, it solves TSP20, TSP50, and TSP100 instances by sampling 1,024 candidates in 17.732 seconds, 41.050 seconds, and 82.561 seconds, respectively. In contrast, OR Tools with 1,024 samples requires significantly longer execution time of 20.604 seconds, 163.074 seconds, and 803.508 seconds for the same problem sizes, exhibiting lower computational efficiency. The execution times reported for Bello et al., Joshi et al., and Costa et al. [45] are derived from their respective studies and are obtained using different hardware platforms. Although this prevents a direct and fair comparison, the substantial time differences remain noteworthy. Even accounting for platform variability, the observed discrepancies suggest that the proposed model achieves a significant advance in computational efficiency.

The experimental results presented in Table 1 and Table 2 clearly illustrate the superior and consistent performance of the proposed model using both greedy and sampling decoding strategies to address the challenges posed by the TSP of varying sizes, establishing it as a robust and effective approach for tackling TSPs.

### Comparison with transformer-based methods

The primary focus of this study is to enhance the efficacy of solving TSP using the Transformer model. As a result, studies employing the Transformer architecture, such as those proposed by Deudon et al. [16], Kool et al. [19], and Bresson et al. [23], serve as particularly pertinent benchmarks owing to their structural and training paradigm similarities. To ensure a fair comparison and minimize performance disparities due to differences in hardware, software platforms, data quantity, and training duration, all models are retrained under identical conditions and evaluated on the same test datasets as our proposed model. Each model undergoes training for 200 epochs. To conduct a comprehensive performance comparison, we test the final models under both greedy decoding and sampling decoding on our benchmark test datasets. Additionally, to facilitate an equitable assessment of the runtime, irrespective of hardware acceleration factors such as CPU, GPU, and parallel computation, each final model and the Concorde are performed on an Intel Xeon Platinum 8255C (2.50 GHz) CPU in a single-threaded context. The runtime for both Concorde and greedy decoding is reported as the average runtime across 10,000 test instances, while for sampling, it is calculated based on the average of 50 instances. The test outcomes of the final models are summarized in Table 3.

**Table 3:** Performance comparison of Transformer-based models on TSP20, TSP50 and TSP100. Obj represents the objective, which refers to the tour length. The optimality gaps (%) are computed with respect to Concorde.

	Method	TSP20			TSP50			TSP100		
		Obj.	Gap (%)	Time (s)	Obj.	Gap (%)	Time (s)	Obj.	Gap (%)	Time (s)
	*Concorde*	3.836	0.00	0.022	5.696	0.00	0.057	7.765	0.00	0.290
*Greedy*	*Deudon et al.*	3.852	0.42	**0.013**	5.830	2.35	**0.029**	8.297	6.85	**0.059**
	*Kool et al.*	3.844	0.21	0.014	5.770	1.30	0.035	8.082	4.08	0.077
	*Bresson et al.*	3.841	0.13	0.030	5.744	0.84	0.076	7.970	2.64	0.163
	*Propsed model*	3.840	0.10	0.016	5.742	0.81	0.037	7.976	2.72	0.076
*Sampling (1,024)*	*Deudon et al.*	3.839	0.08	14.585	5.737	0.72	31.471	8.098	4.29	62.982
	*Kool et al.*	3.838	0.05	14.998	5.717	0.37	39.518	7.908	1.84	81.605
	*Bresson et al.*	3.838	0.05	33.872	5.708	0.21	80.386	**7.850**	**1.09**	170.149
	*Proposed model*	**3.837**	**0.03**	17.732	**5.705**	**0.16**	41.050	7.853	1.13	82.561

Note: The decoding strategies for the comparative models are summarized below. Deudon et al.: (last 3 visited nodes)+pointer mechanism; Kool et al.: (start node, last visited node, graph token) + MHA (unvisited nodes) + Glimpse (unvisited nodes) + SHA (unvisited nodes); Bresson et al.: (last visited node) + MHA (visited nodes) + MHA (unvisited nodes) + SHA (unvisited nodes); Proposed model: (start node, last visited node, all visited nodes) + MHA (unvisited nodes) + Glimpse (unvisited nodes) + SHA (unvisited nodes). The “Glimpse” refers to the glimpse mechanisms inspired by Bello et al. [8]

From Table 3, it can be observed that our model surpasses both Deudon et al.’s [16] and Kool et al.’s [19] models across all three TSP problems in terms of solution quality. This superiority becomes more pronounced as the problem size increases. Employing greedy decoding, our model achieves optimality gaps of 0.10%, 0.81%, and 2.72% for TSP20, TSP50, and TSP100, respectively. Relative to Deudon et al.’s model, our reductions in the optimality gap are 0.32%, 1.54%, and 4.13% for TSP20, TSP50, and TSP100. Similarly, compared to Kool et al.’s model, the reductions of the optimality gap are 0.11%, 0.49%, and 1.36%. In comparison with the state-of-the-art model proposed by Bresson et al., both models exhibit comparable performance, exhibiting only marginal difference. While Bresson et al.’s model shows superior performance on TSP100, our model slightly outperforms Bresson et al. on TSP20 and TSP50 in terms of the optimality gap. The application of sampling decoding further improves the solution quality of each model to varying degrees. Under the sampling mode, our model markedly reduces the optimality gap to 0.03%, 0.16%, and 1.13% for TSP20, TSP50, and TSP100, maintaining a consistent advantage over Deudon et al.’s and Kool et al.’s models across all problems, while also demonstrating comparability with Bresson et al.’s model. Therefore, these results substantiate the effectiveness and competitiveness of our model in proficiently solving TSP.

We conduct a comprehensive analysis of the runtime efficiency of our approach. As illustrated in Table 3, when operating under the greedy decoding mode, our model exhibits remarkable efficiency, requiring only 0.016, 0.037, and 0.076 seconds on average to solve a single instance of TSP20, TSP50, and TSP100, respectively. This performance surpasses that of Concorde and Bresson et al.’s model, and closely aligns with Kool et al.’s model. Our model preserves the rapid problem-solving capability inherent in end-to-end models. Upon transitioning to the sampling decoding mode, our model accumulates a total time cost of 17.732, 41.050, and 82.561 seconds for sampling 1,024 solutions for each problem instance, respectively. This time expenditure is significantly lower than that of Bresson et al.’s model. While the running time of our model is slightly higher compared to Deudon et al.’s and Kool et al.’s models, the solution quality is noticeably improved. The additional time consumption can be attributed to the incorporation of spatial encoding and centrality encoding in the encoder, introducing a degree of complexity and computational overhead. It is noteworthy that while Deudon et al.’s model exhibits the highest execution efficiency, its optimization performance falls significantly short compared to our proposed model. In contrast, Bresson et al.’s model achieves state-of-the-art optimality gaps on TSP100. However, this advancement is achieved through an increased number of attention calculation layers in the decoder phase, leading to a processing time of 170.149 seconds, which is more than double the 82.561 seconds consumed by our model. Therefore, our model demonstrates an outstanding balance between time efficiency and solution quality.

**Fig 5:**
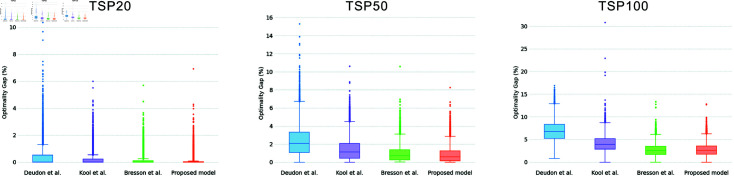
Distribution of optimality gaps of the proposed and comparative models on TSP20, TSP50, and TSP100.

This study additionally compares the stability of Transformer-based models concerning solution quality. Employing greedy decoding, the four models are tested with identical test datasets (size = 10,000) on TSP20, TSP50, and TSP100. The results generated by Concorde are adopted as the benchmark solutions, against which the optimality gap (in percentage) is calculated for each solution. Fig 5 offers a visual representation of the distribution of optimality gaps and Table 4 provides the specific statistics, including the first quartile (Q1), the second quartile (Q2) (also known as the median), and the third quartile (Q3), along with the interquartile range (IQR). The IQR represents the span between Q3 and Q1, delineating the range within which the central 50% of the data resides.

**Table 4:** Quantitative analysis of the optimality gaps (%) between the proposed model and comparative models on TSP20, TSP50, and TSP100

Method	TSP20				TSP50				TSP100			
	Q1	Q2	Q3	IQR	Q1	Q2	Q3	IQR	Q1	Q2	Q3	IQR
*Deudon et al.*	0.00	0.01	0.52	0.52	1.08	2.06	3.33	2.25	5.25	6.73	8.31	3.07
*Kool et al.*	0.00	0.00	0.22	0.21	0.45	1.13	2.07	1.61	2.85	3.96	5.20	2.35
*Bresson et al.*	0.00	0.00	0.10	0.10	0.25	0.71	1.40	1.14	**1.68**	**2.53**	**3.46**	**1.78**
*Proposed model*	**0.00**	**0.00**	**0.03**	**0.03**	**0.16**	**0.58**	**1.24**	**1.08**	1.74	2.60	3.56	1.81

Fig 5 clearly displays that the proposed model consistently exhibits a superior and tight distribution of optimality gaps across all three scales of TSP, surpassing the performance of Deudon et al. and Kool et al. by a significant margin. The superiority becomes more evident with increasing problem size. Referring to Table 4, the Q1, Q2, Q3 and IQR values of the proposed model in TSP20 are equal to or highly close to 0%. This indicates that our model achieves a very high and stable solution quality on small-scale problems, with at least half of the solutions equaling the quality of those generated by the Concorde solver. For TSP50, the quartile values of the proposed model are as follows: Q1=0.16%, Q2=0.58%, and Q3=1.24%. These values imply that when using greedy mode to solve a problem just once, our model has a 25% chance of approximating the optimal solution within a 0.16% gap, a 50% probability of keeping the solution within a 0.58% gap from the optimal, and 75% assurance of maintaining the optimality gap within 1.24%. This demonstrates the high reliability of our model’s solutions. In the case of TSP100, the Q1, Q2, and Q3 values are 1.74%, 2.60%, and 3.56%, with a slight widening of the optimality gap as the problem size increases. However, compared to Deudon et al. and Kool et al., our model exhibits markedly lower quartile values. Moreover, the IQRs of TSP50 and TSP100 are 1.08% and 1.81%, respectively, indicating that the fluctuation in the quality of the majority solutions is small. Additionally, from Fig 5 and Table 4, it can be observed that the distribution of the optimality gaps of our model’s solutions is slightly better than that of Bresson et al. on TSP20 and TSP50. On TSP100, the distribution of the optimality gap of our model’s solutions is comparable to that of the Bresson et al. These statistical findings illustrate that the proposed model is able to maintain remarkable stability in delivering reliable solutions across problems of various sizes.

### Ablation study

**Fig 6:**
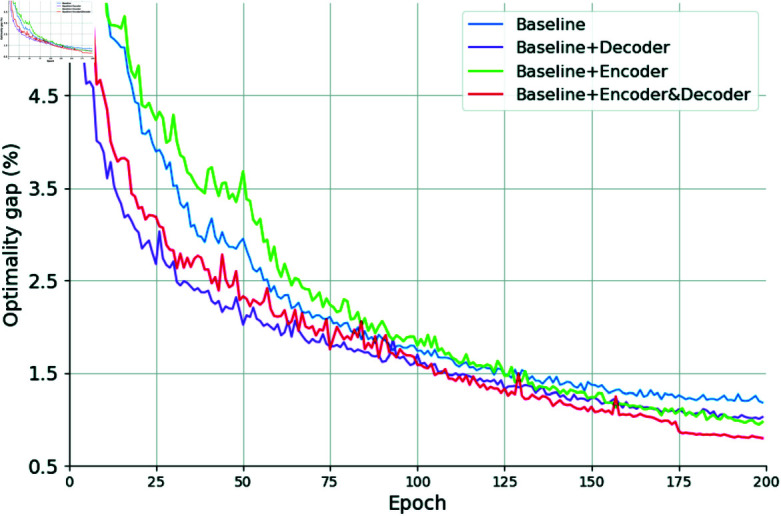
Performances of diverse configurations of the proposed model throughout training on TSP50.

To illustrate the influence of the proposed encoding and decoding mechanisms on model performance, we conduct a comprehensive ablation study on the TSP50 dataset. Multiple model variants, each incorporating different subsets of the proposed enhancements, are trained under identical hyperparameter configurations and computational conditions. Their performance is evaluated on a fixed validation set of 10,000 instances, using percentage-wise optimality gaps relative to Concorde. The results, depicted in Fig 6, demonstrate that both the encoding and decoding mechanisms independently improve performance, and their combined integration yields the greatest overall improvement.

Starting with the baseline model (blue curve), we observe a moderate convergence rate and a certain steady-state optimality gap. By introducing the proposed encoding mechanism—incorporating spatial encodings to capture relational information among nodes and centrality encodings to reflect underlying graph-theoretic importance—we obtain a model (green curve) that learns richer node representations. Although this enhancement initially slows early-stage training due to increased model complexity, it ultimately facilitates better discernment of global and local structural patterns, enabling the model to identify higher-quality tours. This improvement underscores the value of our encoding for transforming raw TSP data into more semantically rich and geometrically meaningful representations.

Similarly, augmenting the baseline with our decoding mechanism (purple curve) leads to substantial performance improvements. Grounded in a reinforcement learning framework, the decoder models the TSP as a Markov Decision Process, where decisions about the next node are guided by a state representation that reflects the current progress of the tour. By incorporating the entire set of visited nodes as a crucial component, the decoder gains a more accurate and holistic view of the decision context, enabling it to make better-informed decisions. This approach aligns with the theoretical foundations of the Held-Karp [46] algorithm, which ensures global optimality by considering subsets of visited nodes. By leveraging this enriched decoding context, instead of relying solely on limited local information, such as the last visited node or a narrow window of recent nodes, the decoder achieves faster convergence and significantly reduces the final optimality gap compared to the baseline. These results underscore the ability of the decoder to more effectively approximate globally optimal solutions and highlight the importance of integrating comprehensive state representations into the decision-making process.

The most pronounced improvement emerges when both the enhanced encoder and the globally informed decoder are integrated (red curve). In this configuration, the model benefits simultaneously from comprehensive structural representations and globally coherent decision-making, accelerating convergence and achieving the smallest overall optimality gap. This synergy suggests that the superior performance of the full model is not attributable to a single innovation, but rather to the complementary effects of both the encoding and decoding strategies.

### Scalability and limitations

Scalability is a pivotal aspect of any algorithm in combinatorial optimization, yet scaling end-to-end approaches to more complex, realistic instances remains an open problem [1,28,38,39,47,48]. Training learning-based models on large-scale graphs is computationally intensive, and models trained solely on small-scale datasets often struggle to larger instances due to distributional changes [39,47,49,50]. For example, Joshi et al. [47] reported that even after training directly on 12.8 million TSP200 samples for more than 500 hours, their learning-based model did not outperform a simple insertion heuristic, underscoring the difficulty of scaling to larger problem sizes.

The Transformer architecture, with its quadratic complexity, further intensifies these computational challenges by substantially increasing both the memory usage and the demand for extensive data. In our own attempts, training a TSP200 model with a batch size of 512 under our experimental setting required approximately 53GB of GPU memory, which significantly exceeds the capacity of typical academic-scale hardware. Even using an NVIDIA A800 GPU, the training process required approximately 40 minutes per epoch, and progress became minimal after 28 epochs, prompting early stopping. Notably, other closely related studies [16,19,23] also restrict their models to TSP100. These findings underscore the prohibitive nature of fully training models on larger instances with typical hardware, reflecting a widely recognized scalability barrier encountered by current Transformer-based approaches.

Despite these challenges, evaluating the scalability of the proposed model under more demanding and practical conditions remains crucial for understanding its strengths and limitations. To this end, we tested our medium-scale TSP50 model on a diverse set of TSPLIB instances. These instances vary considerably in size, ranging from approximately 1 ×  to 4 ×  the default model size, thereby allowing us to gain insights into the model’s performance across a broad range of scenarios. For each instance, we adopted a sampling mechanism (1,024) to generate the solution, and the results are presented in Table 5.

**Table 5:** Testing results on TSPLIB instances using the TSP50 model for assessing the scalability of the proposed approach. The table includes optimal values for reference, with the best performance among the four tested algorithms highlighted in bold. Performance gaps (%) indicate deviations from the optimal value.

Instance	Optimal	Deudon et al.		Kool et al.		Bresson et al.		Proposed model	
		Obj.	Gap (%)	Obj.	Gap (%)	Obj.	Gap (%)	Obj.	Gap (%)
*eil51*	*426*	453	6.34	430	0.94	428	0.47	**427**	**0.23**
*eil76*	*538*	573	6.51	558	3.72	**550**	**2.23**	552	2.60
*eil101*	*629*	676	7.47	654	3.97	**643**	**2.23**	689	9.54
*pr76*	*108,159*	111,449	3.04	111,033	2.66	112,432	3.95	**110,911**	**2.54**
*pr124*	*59,030*	61,335	3.90	**61,079**	**3.47**	61,362	3.95	62,177	5.33
*pr226*	*80,369*	**85,506**	**6.39**	95,823	19.23	99,886	24.28	94,191	17.20

The experimental results demonstrate that the proposed model performs well on small-scale problems comparable in size to the pre-trained model, achieving the lowest gaps among the comparative algorithms on eil51 (0.23%) and pr76 (2.54%). It also performs competitively on eil76, with a gap of 2.60% slightly trailing Bresson et al.’s 2.23%. Despite being trained solely on randomly generated data, our model performs robustly on these instances, suggesting its potential for addressing small-scale applications, such as logistics optimization for regional delivery routes and vehicle routing for local transportation networks. However, when the problem size doubles, as in the cases of eil101 and pr124, the model’s performance shows a noticeable decline. When the problem size scales to around four times the default model size, the decline becomes significant, with the gap expanding to 17.20% on pr226, encountering significant scalability limitations. Notably, these limitations are consistent across the comparative models, such as those of Kool et al. and Bresson et al. on pr226. Moreover, none of the models exhibits consistent dominance in these test instances. These findings reaffirm that scalability remains a pervasive challenge in the field of neural combinatorial optimization.

The commendable performance of the proposed model on small-scale problems can be attributed to the effective integration of spatial and centrality encodings, which introduce beneficial biases in how the Transformer attends to the input. For smaller problems, these encodings emphasize critical features, such as the centrality of a city or local spatial relationships, guiding the model’s attention to important parts of the problem. However, larger-scale TSPs often feature more uniform node distributions or complex, long-range interactions that are not as effectively captured by the same encodings. These biases, which are advantageous for small instances, may mislead the model at scale by overemphasizing features that become less informative or even counterproductive in larger graphs. Adaptive encoding schemes that dynamically adjust to problem size and data distribution, along with transfer learning and data augmentation, are promising approaches to address these scalability challenges.

## Conclusion

End-to-end models built upon the Transformer have become a research hotspot in neural combinatorial optimization due to their outstanding efficacy. This study explores how to integrate graph structure information into the Transformer framework to further enhance the performance of neural combinatorial optimization and proposes a structure-aware model specifically for solving TSP. Through the additional closeness centrality encoding and spatial encoding, the proposed model augments Transformer’s capacity to perceive the graph structure inherent in TSP instances, especially for smaller-scale problems. Furthermore, this study introduces a modified decoding mechanism that not only emphasizes the starting node and the previously visited node, but also captures the dynamic evolution of the tour by covering all the nodes that have been visited so far. This design provides the decoder with more informed decision states for making better predictions and ultimately improves the overall performance of the model. Trained through deep reinforcement learning, the proposed model achieves results closely approaching optimality for 2D Euclidean TSP instances with up to 100 nodes, surpassing various comparable models in terms of solution quality. Coupled with its rapid solving speed and robust stability, it shows an outstanding balance between efficiency and effectiveness, positioning it as a competitive choice for tackling TSP. This study demonstrates the positive influence of graph structure information in addressing TSP. Additionally, it serves as a showcase for developing effective improvement mechanisms to leverage this valuable information, thereby enhancing the performance of neural combinatorial optimization models based on Transformer.

Scalability is a common issue in the field of neural combinatorial optimization, particularly for large problem instances and practical applications. As discussed previously, the encoding and decoding mechanisms play a critical role in determining the performance of the model. Consequently, designing more effective encoding and decoding mechanisms to enhance the model’s scalability across various problem scales warrants further exploration. Additionally, model training requires substantial computational resources, which limits the model’s ability to efficiently learn from large-scale instances. Approaches such as sparse attention mechanisms and hierarchical models show promise in reducing computational complexity, making them valuable directions for future investigation. For applications to practical benchmark instances, such as those in the TSPLIB benchmark, the model performance often lags behind its success on synthetic datasets. Techniques like fine-tuning and domain adaptation could improve generalization to these applications. However, effective strategies for adapting the model to such instances remain underexplored and represent an important avenue for future research.

## Supporting information

Data The accompanying ZIP package includes data used in this research. (ZIP)
